# Habitat use of bats in relation to wind turbines revealed by GPS tracking

**DOI:** 10.1038/srep28961

**Published:** 2016-07-04

**Authors:** Manuel Roeleke, Torsten Blohm, Stephanie Kramer-Schadt, Yossi Yovel, Christian C. Voigt

**Affiliations:** 1 Department of Evolutionary Ecology, Leibniz Institute for Zoo and Wildlife Research, Berlin 10315, Germany; 2 Dorfstraße 48, Prenzlau 17291, Germany; 3 Department of Zoology, Faculty of Life Sciences, Tel Aviv University, Tel Aviv 6997801, Israel

## Abstract

Worldwide, many countries aim at countering global climate change by promoting renewable energy. Yet, recent studies highlight that so-called green energy, such as wind energy, may come at environmental costs, for example when wind turbines kill birds and bats. Using miniaturized GPS loggers, we studied how an open-space foraging bat with high collision risk with wind turbines, the common noctule *Nyctalus noctula* (Schreber, 1774), interacts with wind turbines. We compared actual flight trajectories to correlated random walks to identify habitat variables explaining the movements of bats. Both sexes preferred wetlands but used conventionally managed cropland less than expected based on availability. During midsummer, females traversed the land on relatively long flight paths and repeatedly came close to wind turbines. Their flight heights above ground suggested a high risk of colliding with wind turbines. In contrast, males recorded in early summer commuted straight between roosts and foraging areas and overall flew lower than the operating range of most turbine blades, suggesting a lower collision risk. Flight heights of bats suggest that during summer the risk of collision with wind turbines was high for most studied bats at the majority of currently installed wind turbines. For siting of wind parks, preferred bat habitats and commuting routes should be identified and avoided.

Over the past two decades, wind turbines have been installed in large numbers worldwide. Wind energy production is heavily promoted in Europe and especially in Germany, owing to the so-called ‘Energiewende’, which is a full transition of power production from fossil and nuclear sources to renewable energy sources. As a result, Germany ranks third worldwide with respect to total net energy production from wind power and second in density of wind turbines^1^,^2^,^3^. Meanwhile, evidence accumulates from many countries that large numbers of bats are killed by wind turbines^4^,^5^,^6^,^7^. Recent studies suggest that wind turbines might attract bats^8^,^9^. Although mitigation measures, such as increased cut-in speeds, might be considered when local conditions favour bat activity^10^,^11^, these measures might not necessarily be efficient for reducing bat fatalities^12^.

The development of efficient mitigation measures also suffers from the difficulty in studying the spatial behaviour of a taxon that is highly elusive and mobile at the same time. Previous studies on the foraging behaviour of bats used mostly conventional VHF radio-tracking, since conventional GPS devices were too heavy^13^,^14^,^15^,^16^,^17^. Recently, miniaturized GPS devices became available, which enables current researchers to record the foraging behaviour of even small to medium-sized bats at high spatial resolution^18^. Here, we used such devices to track the movements of common noctule bats, *Nyctalus noctula* (Schreber, 1774), in north-eastern Germany, an area characterized by farmland that is heavily used for both agriculture and wind power production.

*Nyctalus noctula* belongs to the functional group of so-called open-space foraging bats^19^ which hunt insects in uncluttered habitats and relatively high above ground, and thus possibly in the vicinity of operating rotor blades. Indeed, *N. noctula* make up the majority of bat fatalities at wind turbines in Germany^20^. Lehnert *et al.*^21^ recently found that about 28% of *N. noctula* killed by wind turbines originated from distant places such as Poland, Baltic countries and Belarus, whereas the majority (72%) were of regional origin. This emphasizes the need to better understand how local bat populations respond to wind turbines.

Here, we used miniaturized GPS loggers to record the fine scale movements of adult male and female *N. noctula* between May and July, when recently weaned juveniles begin to disperse from their maternity roosts. We expected that movement patterns will vary between males recorded in early summer and females recorded in midsummer due to different life history stages. Assuming that insect abundance was highest above wetlands and grasslands, we also expected that foraging *N. noctula* will prefer these habitats over others. Furthermore we tested if *N. noctula* are attracted by visible prominent structures such as wind turbines and linear structures such as hedgerows and treelines.

## Results

### Movement behaviour

**Figure 1:**
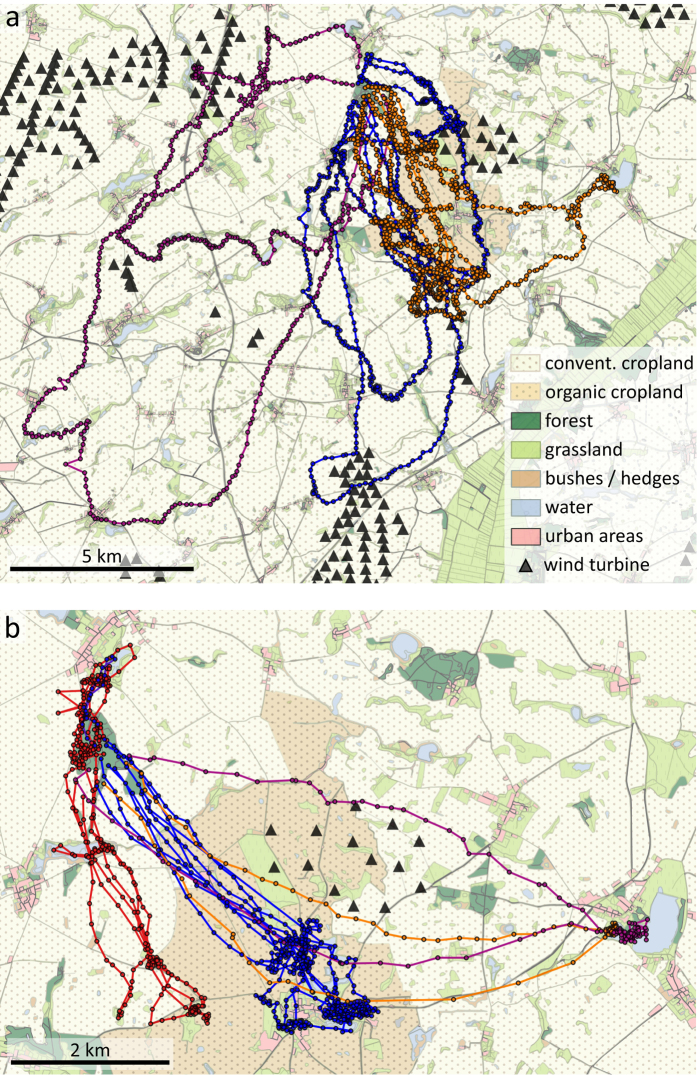
Evening flight paths of the three female (a) and five male (b) *N. noctula*. Different colours depict different individuals. For most individuals we recorded multiple paths throughout several nights. Maps were created with ArcGIS Desktop 10.2 (ESRI Inc, USA, http://www.esri.com/apps/products/download/) using data from^39^,^40^.

**Figure 2:**
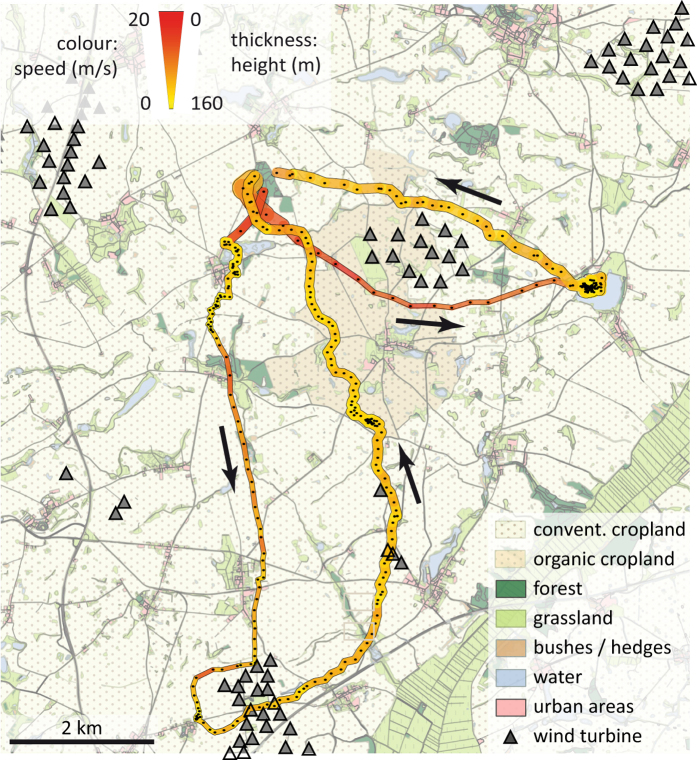
Exemplary evening flight paths of a female (lower path) and male (right path) *N. noctula*. Colour and thickness of paths code for flight speed and flight height. Black dots within paths depict recorded GPS locations. Black arrows depict the overall flight direction. The map was created with ArcGIS Desktop 10.2 (ESRI Inc, USA, http://www.esri.com/apps/products/download/) using data from^39^,^40^.

Evening trips of female (n = 3) and male (n = 5) *N. noctula* differed in duration (U = 15, p = 0.04, n = 8) and distance travelled (U = 15, p = 0.04, n = 8), yet we note that females and males were recorded at different months. On average, the emergence time was 24 ± 15 min after sunset. Evening trips of female *N. noctula* lasted on average 105 ± 20 min and covered an average distance of 26.6 ± 4.6 km, whereas trips of males lasted only 61 ± 32 min and covered a distance of only 14.6 ± 7.4 km (see Fig. 1 for all tracks). The farthest distance from the roost that individuals reached did not differ between sexes (U = 13, p = 0.14, n = 8) (Supplementary Material Fig. S1). On average, the farthest point from the roost averaged 5.8 ± 2.9 km for all bats, yet we recorded one female as far as 13.7 km from the roost. For all evening trips of the three females, 95% of GPS locations lay within a distance of 6.9, 9.3, and 12.9 km from the roost, respectively (Supplementary Material Fig. S2). Comparing the speed of bats when traversing cropland, we found that male *N. noctula* flew almost 1.5 times faster than females (males: 6.2 ± 1.8 m/s, females: 4.2 ± 0.2 m/s, U = 15, p = 0.036, n = 8) (Fig. 2). During evening trips, females flew higher than males when above cropland or grassland (females: 64 ± 1 m, males: 35 ± 18 m, U = 15, p = 0.036, n = 8). Three females and three males occasionally also performed short trips in the morning, likely in order to drink at nearby water bodies. These morning trips covered distances of on average 5.8 ± 3.0 km during a mean trip duration of 22 ± 12 min. The morning trips ended on average 25 ± 12 min before sunrise. Duration and travelled distance of these morning trips did not differ between sexes (duration: U = 6.5, p = 0.51, n = 6, travelled distance: U = 6, p = 0.70, n = 6).

### Resource use

**Figure 3:**
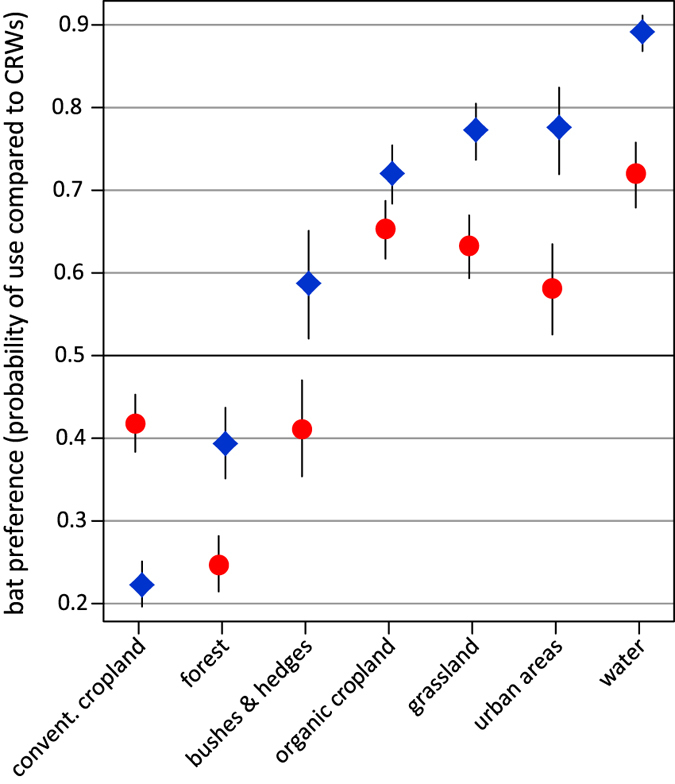
Probability of bat presence for the respective land use categories. Probability values greater than 0.5 indicate that it was more likely to find bats, values smaller 0.5 indicate that it was less likely to find bats in the respective habitat based on the availability of the habitat. Red points represent data from female, blue squares represent data from male *N. noctula*. Whiskers represent 95% confidence intervals.

**Figure 4:**
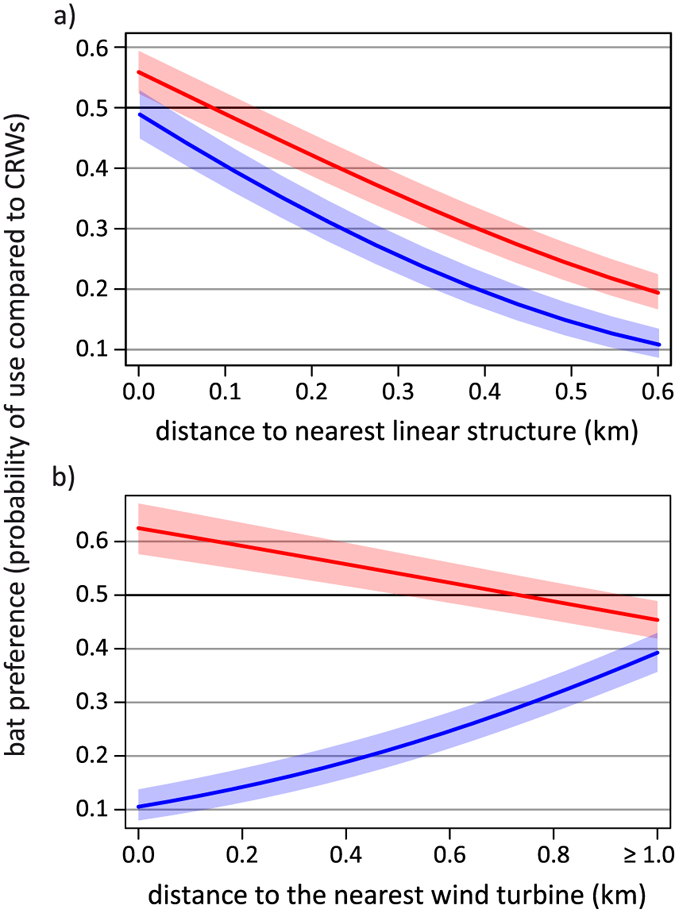
Probability of bat presence in relation to distance towards linear structures (a) and distance towards wind turbines (b). Probability values greater than 0.5 indicate that it was more likely to find bats, values smaller 0.5 indicate that it was less likely to find bats at the respective distance. The red lines represents data from female and the blue lines those from male *N. noctula*. Bands show the 95% confidence intervals.

Land use type, distance to wind turbines, distance to linear structures such as treelines and hedgerows, and the interactions of these variables with sex/season all contributed significantly to explain the presence of bats (Supplementary Material Table S1). We refer to this latter variable as sex hereafter, although we cannot exclude seasonal effects within this variable, owing to our recording schedule (males in early summer, females in midsummer). Female *N. noctula* used open water more often than expected from correlated random walks (CRW), followed by organic cropland, grassland, and urban areas. We found that more than 54% of their GPS locations lay above conventional cropland. However, this is still less than expected based on availability. Forest was mostly avoided by females (Fig. 3). Male *N. noctula* also preferred open water, followed by urban areas, grassland, and organic cropland. With only about 21% of GPS locations above conventional cropland, males used this habitat less often than females (Fig. 3). Both sexes flew close to linear structures more often than expected (Fig. 4a).

**Figure 5:**
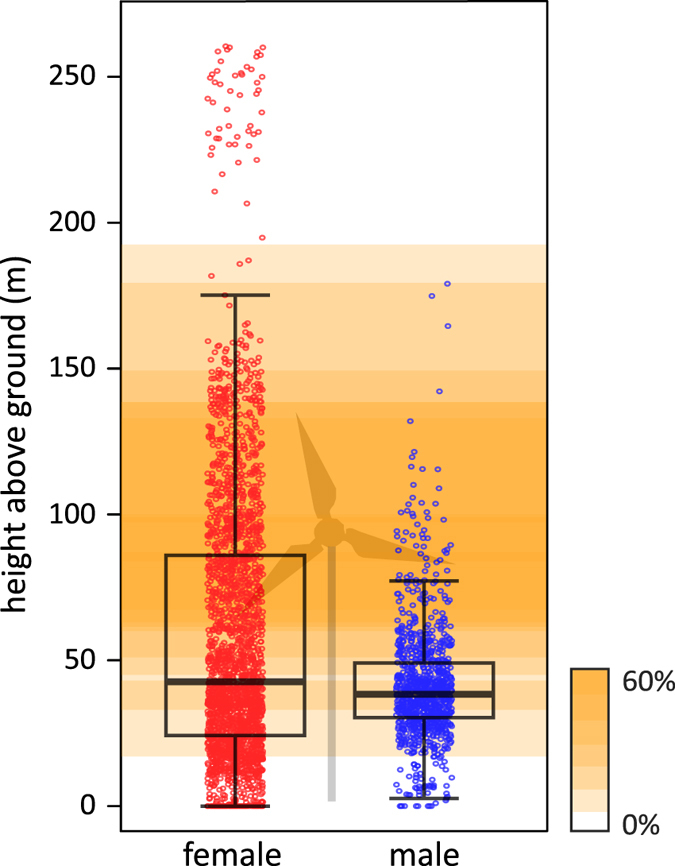
Flight heights of female and male *N. noctula* over open habitats (i.e. cropland and grassland). Intensity of background colour depicts the density of turbine blades in the study area at the respective heights. Dots depict height of single GPS locations, boxes and whiskers depict the quartiles for flight heights of male and female bats, with thick line showing the median flight height.

The relative probability of recording a bat at a given distance to a wind turbine differed between sexes. While we recorded females closer to wind turbines than expected, males seemed to have avoided wind turbines (Fig. 4b). Indeed, we observed that two out of three females crossed large wind parks or performed short foraging bouts less than 100 metres away from wind turbines. We did not observe such behaviour in the five tracked males, nor any attempts to approach turbines. In our study area (i.e. 20 km radius around the roost), the height range of 67 to 133 m is most intensively used for wind power production (60% of turbine blades operate in this area). For male *N. noctula*, 8% (n = 80/1009) of GPS locations over open habitat (i.e. cropland and grasslands) were within this height range, whereas 28% (n = 711/2469) of female GPS locations over open habitat were within this range (Fig. 5). Subsumed for all bats, 95% of GPS locations over open habitat were recorded at heights between 0 and 144 m above ground.

## Discussion

Using miniaturized GPS devices, we monitored the movement patterns of eight *N. noctula* above agricultural land that is intensively used for wind power production. We found that males made relatively short commuting trips to their well-defined foraging grounds during early summer. During midsummer, females made larger journeys, traversing the landscape with relatively low flight speed. On some of these trips, females came close to wind parks, crossed lines of wind turbines or foraged in direct proximity to wind turbines (i.e. less than 100 m from the turbine poles). Males on the other hand neither foraged close to wind turbines nor crossed wind parks. We observed possible avoidance of wind turbines in two out of the five males which made detours around a large wind park rather than crossing it in direct line. When commuting over open landscapes, females often flew at heights above ground that were intensively used for wind power production. Male and female *N. noctula* both preferred water bodies and adjacent urban areas, grassland, and organic cropland. In contrast, they used conventional managed cropland less often than expected from availability. Preference patterns were stronger for males recorded in early summer than for females recorded in midsummer. Since we encountered a low retrieval rate in midsummer, we could not obtain any data from male bats for this season. Thus, we cannot say if the detected differences can be attributed solely to sex-specific differences, to seasonal variation, or a combination of both. However, Blohm^13^ reported that female *N. noctula* from the study area begin to leave their summer roosts in August, probably searching for mating opportunities in a wide area around the initial roost. Thus the large flight trips we recorded here might have depicted the onset of this change in behaviour, suggesting that the differences we found were indeed an interaction of the factors sex and season.

Analysis of general movement patterns showed that females travelled over distances almost twice as long as males. Females also spent about twice as much time airborne compared with males. Females regularly traversed the landscape in extended journeys, most often using different flight paths to preferred foraging habitats. The maximum distance of a female to its roost was about 14 km. In combination with the observation that females flew slower above cropland than males, these findings lead us to conclude that females use a different foraging tactic in midsummer than males in early summer. While males followed a daily routine of commuting to well defined areas for intensive foraging, females probably fed en route. We speculate that motivations and constraints were different for males and females when choosing specific flight paths. Females may have searched for additional foraging grounds, social partners or alternative roosts for mating in midsummer. In contrast, males probably choose familiar foraging grounds that were relatively close to their roosts, which seems most efficient when assuming that prey availability at foraging grounds was predictable.

Both sexes used the available habitats in a non-random manner. Water bodies were mostly preferred, which is consistent with several studies that used mostly acoustical monitoring^22^,^23^,^24^, yet this finding contrasts with stable isotope data from *N. noctula* killed during the autumn migration period by wind turbines^25^. Furthermore, *N. noctula* preferred to fly above grassland, which is also in line with several other studies^26^ (for pasture^24^, for forest-farmland ecotone). Another habitat category that was extensively used by the bats was urban areas. However, the two small settlements above which we recorded the highest activity of *N. noctula* were both associated with small lakes and grasslands, which might explain the preference for urban areas merely as a confounding effect of this proximity. Still, observations of the trajectories revealed that the bats preferentially flew in areas with both elements: lakes and urban areas. This suggests that the contact zone between settlements and lakes may be most attractive for *N. noctula*. The preference for this contact zone might also explain why *N. noctula* were often found close to linear structures, since hedges and roads are often associated with water bodies and urban environments. Artificial lighting and complex vegetation structure within human settlements possibly attracted many insects with aquatic larval stages, and thus could provide ideal food resources for *N. noctula* in these areas. *N. noctula* foraging around street lamps have already been observed before by Kronwitter^16^ and Rydell^27^. We also observed foraging of *N. noctula* close to linear structures, such as vegetation accompanied roads, treelines and hedges. These structures were possibly preferred habitats for prey insects^28^, thus providing foraging grounds for open space foragers like *N. noctula*. Possibly, the bats used linear structures in open landscapes for orientation, as is suggested for pigeons in similar landscapes^29^,^30^.

Conventional cropland on the other hand was used less often than expected, although this is the predominant land use type and offers the largest open space in the Uckermark area. Mackie and Racey^26^ and Ciechanowski^31^ also observed relatively low activity of *N. noctula* above cropland. This indicates that cropland offers little food resources for *N. noctula*. Yet, our data suggests that bats might successfully forage over organic cropland. Since all organic managed fields in our study area were clustered around a single village and are thus spatially auto-correlated, our data are insufficient to draw any final conclusions on preference of *N. noctula* for organic cropland. The fact that all habitat preferences were less pronounced for female than for male *N. noctula* is consistent with our assumption of sex- and season-specific foraging strategies.

Movement behaviour of *N. noctula* in open space is of particular interest in relation to their high risk of colliding with wind turbines^6^, since wind turbines are most often erected in open areas. Recently, Voigt *et al.*^12^ estimated that more than 250,000 bats might be killed per year by wind turbines in Germany when no mitigation measures are applied. In the region of our study, *N. noctula* accounts for the majority of all bat fatalities (49%^20^) and most fatalities in Germany occur between August and September. This coincides with the mating and migration season of *N. noctula*^32^,^33^, but is also the time during which we observed the extended foraging journeys of female *N. noctula*. In accordance with our observations of females repeatedly flying close to wind turbines, we assume that also resident *N. noctula* are likely killed by wind turbines in the Uckermark. This is supported by a study of Lehnert *et al.*^21^ showing that 72% of *N. noctula* killed by wind turbines in Eastern Germany originated from local or regional populations, and that most of the adult fatalities were females (64%). Our observation that males did not frequently interact with wind turbines might be due to the fact that males already had established fixed routes to their daily foraging grounds. We speculate that wind turbines might be still dangerous for juvenile males when establishing such routes for the first time. A study in Saxony, Germany^34^ supports this suggestion, finding that almost exclusively juvenile *N. noctula* of both sexes were killed by wind turbines. However, a likely avoidance of wind turbines which we observed in the commuting routes of two males suggests that wind turbines may alter the habitat use of *N. noctula*. This implies that large wind parks might constrain daily commuting routes and thus disconnect potential feeding from roosting sites, leading to habitat loss for bats.

While the strict flight routine of male *N. noctula* explains why they rarely came close to wind turbines, it remains unclear why female *N. noctula* flew closer to wind turbines than expected. Bats approaching wind turbines have been described before. Horn *et al.*^35^ and Cryan *et al.*^9^ observed bats exploring wind turbines, possibly in search for roosts. Cryan^36^ considered that tree-roosting bats, like *N. noctula*, mistake wind turbines for tall trees and establish mating territories around them. Rydell *et al.*^37^ on the other hand state that bats are most likely foraging around wind turbines because these may attract insects and thus offer food resources to bats. Ahlen *et al.*^38^ noticed especially *N. noctula* foraging around offshore wind turbines. Indeed, in two cases we observed female *N. noctula* foraging for several minutes only a few hundred metres away from wind turbines. On another occasion, we recorded a female approaching a wind park and finally crossing it, and only some minutes later crossing another line of wind turbines (lower part of the lower track in Fig. 1). All of these manoeuvres were performed at heights at which rotor blades were operating. Assuming an attraction towards the wind turbine, it might be possible that the female was inspecting the turbine as a potential roosting site. However, since the bat did not reduce flight speed during these manoeuvres, she might have also used the wind park as a landmark for orientation. Our measurements of flight heights of bats over open habitats emphasize the potential conflict between foraging bats and wind turbines. Heights between 70 and 130 m were most intensively used for wind power production in our study area. More than one fourth of female GPS locations recorded above open landscape fell into this range. Overall, our observations suggest that wind turbines may constitute a severe threat for *N. noctula*, especially when erected close to preferred foraging habitats like water bodies.

Here, we demonstrated that the fine-scale monitoring of foraging behaviour of individual bats can contribute to our understanding of how bats use anthropogenic landscapes. Although restricted to a relatively small number of individuals, we observed substantial differences in the behaviour of male *N. noctula* in early summer and female *N. noctula* in midsummer. Although both sexes preferred water bodies and used conventional cropland less often than expected from availability, this pattern was more pronounced for male than for female *N. noctula*. Females used areas at least as far as about 14 km from their roosts. During these long trips, they came repeatedly close to wind turbines. Our data suggests that wind turbines may attract female *N. noctula* in midsummer. Further, females regularly used flight heights above ground that overlapped largely with the operational range of wind turbine blades. Male *N. noctula* on the other hand followed a rigid routine of commuting to their established foraging grounds within a maximum distance of about 7 km, yet they stayed mostly away from wind turbines. The occasionally observed avoidance of wind turbines by adult male *N. noctula* in early summer suggests an exclusion from those habitats encompassed by wind turbines and an interruption of commuting routes between roosts and foraging patches; thus leading eventually to habitat loss. The observed difference in the movement behaviour of females and males is important for predicting the vulnerability of collisions with wind turbines. Our findings demonstrate that at least in midsummer wind turbines may represent a potential threat for local colonies of high flying bat species like *N. noctula*, even within a relatively large distance from roosts. Those habitats preferred by *N. noctula*, such as small water bodies and adjacent areas, as well as flight corridors between roosting sites and preferred foraging habitats, should be avoided for siting of wind turbines. During our study period, bats might have been at risk of colliding especially with wind turbines operating at low heights above ground, which demands mitigation measures such as cut-in speeds especially for these facilities during summer.

## Materials and Methods

### Study site and GPS tracking

The study was conducted in 2014 in the Uckermark area in north-eastern Germany. The study area is dominated by crop farming, numerous wind parks and mainly small and scattered eutrophic water bodies^39^,^40^. The study population of *N. noctula* mainly uses artificial bat boxes in a small isolated forest patch (Carmzower Forst, 53°22.371′N, 14°2.870′E) and has been continuously monitored for 20 years^41^. In 2014, the colony consisted of roughly 500 individuals, including about 200 reproducing females and their offspring. We attached miniaturized GPS devices (Robin GPS Loggers, CellGuide Ltd., Israel) to the back of bats using a combination of custom-built collars and skin glue (Sauer Hautkleber, Manfred Sauer, Germany). GPS devices were programmed to record positions of bats every 30 seconds, starting each day one hour before sunset and lasting until one hour after sunrise. We attached GPS devices around noon. On average, about 30 minutes passed between retrieving bats from bat boxes and returning bats back into their box. Since females were still weaning in early summer, we only tracked males during this time (i.e. May and June), resulting in data for five individuals. In midsummer (i.e. July), we recorded data from three females. Accordingly we obtained data from eight out of 24 tagged individuals. We recorded one to five nights for a given individual (2.9 ± 1.8 nights per individual). In total, we analysed data from 23 nights resulting in 40 continuous bat trips either recorded shortly after sunset or shortly before sunrise (Fig. 1). Hereafter, we refer to these trips as evening or morning trips respectively.

The additional load of the used GPS loggers exceeded the 5% threshold of body mass commonly recommended for birds and bats^42^. However, the authors of that study state that bats might be able to cope with additional loads of up to 30% of their body mass. Cvikel *et al.*^18^ confirmed for the similar-sized *Rinopoma microphyllum* that bats of about 30 g are capable of carrying an additional load of 4 g without any apparent changes in foraging behaviour. To assess if the additional load of the GPS devices had a negative impact on our study bats, we ran a Wilcoxon signed rank test on body mass measured before attaching the GPS device and after removing the device about one week later. The logger mass ranged from 3.4 to 4.2 g or 9.2 to 11.5% of individual body masses of bats, respectively. Comparison of body masses before attachment of the devices (33.9 ± 3.2 g) and after recapture of the animals (32.9 ± 1.7 g) revealed no significant change in body mass (W = 18, p = 0.14, n = 7). Furthermore, large flight trips of female *N. noctula* as well as regular emergence times (cf. ref. 16) lead us to assume that tagged bats exhibited a foraging behaviour similar to untagged individuals. Furthermore, five of the study animals used the roosting boxes also in the following year, suggesting that impact on health as well as disturbance due to the experiments were negligible. Our work was approved by the Landesamt für Umwelt, Gesundheit und Verbraucherschutz Brandenburg (permit: LUGV_RO7-4743/63+4#46841/2014). All used methods were in accordance with this permit and the ASAB/ABS Guidelines for the Use of Animals in Research.

### Movement data

We estimated minimum travel speed as net displacement between two consecutive GPS locations divided by time passed, flight height above ground, duration of flight trips, and total travel distance for each flight trip. Since the number of recorded flight trips varied between individuals, we randomly chose one trip per individual to compare these measures between bats using Mann-Whitney-U tests. Since the on-board recorded flight heights were not reliable when bats used structured habitats (e.g. forest), we only compared flight heights over open habitats (i.e. cropland and grassland). We used an alpha threshold of 5% for all statistical analysis in this work. Means are always given as mean ± standard deviation.

### Resource use analysis

We assigned underlying land use type and location of linear structures (subsumption of roads, alleys, railway tracks, treelines, hedgerows, and flowing waters) and wind turbines to GPS locations using aerial infrared imagery maps^39^,^40^ in ArcGIS Desktop 10.2 (ESRI Inc, California, USA). We compressed land use types into seven categories: conventionally managed cropland, organically managed cropland, grassland, forest, urban area (including also rural areas), open water, and shrub land. We used land use type, distance to wind turbines, and distance to linear structures as environmental predictor variables for presence of bats. To account for variation between females and males recorded in the respective seasons, we included the interaction of all predictor variables with sex/season. To test for habitat preferences, we chose a use vs. availability framework^43^, allowing to compare the resources used by bats with the availability of these resources as derived by simulated random movements. As null model for spatial randomness incorporating movement constraints^44^ we created five correlated random walks (CRWs) for each bat trip^45^ using the R package “adehabitatHR”^46^. Step length and turning angle were independently chosen from the original bat trips and used to create CRWs with sampling frequency identical to that of the corresponding bat trips. To ensure that CRWs lay within the available area of individual bats, we only allowed CRWs within the minimum convex polygon (MCP) of the original trip in addition to a buffer zone which measured one half of the square root of the MCP area (cf. refs 47,48 for usage of half step length). All CRWs started at the roost where bats were tagged.

### Resource use model

To test whether *N. noctula* preferred certain habitats over others, we compared bat trips (presence) to CRWs (pseudo-absence) with a generalized mixed effects model (GLMM) with binominal error structure and logit link, using the R package “lme4”^49^. Before fitting the model we used Pearson’s moment correlation test to ensure that explanatory variables were not correlated (Pearson’s |r| < 0.25). We used land use type, distance to wind turbines, distance to linear structures, and interactions of these variables with sex/season as explanatory variables to explain presence of bats. We used the respective bat trips nested within the individual bat identifier as random factor. We selected the final model by comparison of Akaike’s information criterion (AIC) (Supplementary Material Table S2). We used the R package “effects”^50^ to visualize the impact of the habitat variables (i.e. land use type, distance to nearest wind turbine and distance to nearest linear structure) on the bats’ movement behaviour. Plots show 95% confidence intervals of habitat parameters to facilitate interpretation of resource use.

## Additional Information

**How to cite this article**: Roeleke, M. *et al.* Habitat use of bats in relation to wind turbines revealed by GPS tracking. *Sci. Rep.* **6**, 28961; doi: 10.1038/srep28961 (2016).

## Supplementary Material

Supplementary Information
