# Supplementary material for: Habitat use of bats in relation to wind turbines revealed by GPS tracking
Source: Sci Rep. 2016 Jul 4;6:28961. doi: 10.1038/srep28961 (PMC4931514; doi:10.1038/srep28961)
Supplement: Supplementary Information [file srep28961-s1.pdf]

Habitat use of bats in relation to wind turbines revealed by GPS tracking

Roeleke Manuel[1], Blohm Torsten[2], Kramer-Schadt Stephanie[1], Yovel Yossi[3], Voigt C. Christian[1]

[1]Department of Evolutionary Ecology, Leibniz Institute for Zoo and Wildlife Research, Berlin 10315, Germany

[2] Dorfstraße 48, Prenzlau 17291, Germany

[3] Department of Zoology, Faculty of Life Sciences, Tel Aviv University, Tel Aviv 6997801, Israel

Email addresses

Torsten Blohm: torsten-blohm@t-online.de

Stephanie Kramer-Schadt: kramer@izw-berlin.de

Yossi Yovel: yossiy@post.tau.ac.il

Christian C. Voigt: voigt@izw-berlin.de

Corresponding author: Manuel Roeleke

roeleke@izw-berlin.de

Phone: ++ 49-30-5168-423

Fax: ++ 49-30-5126-104

Supplementary Material

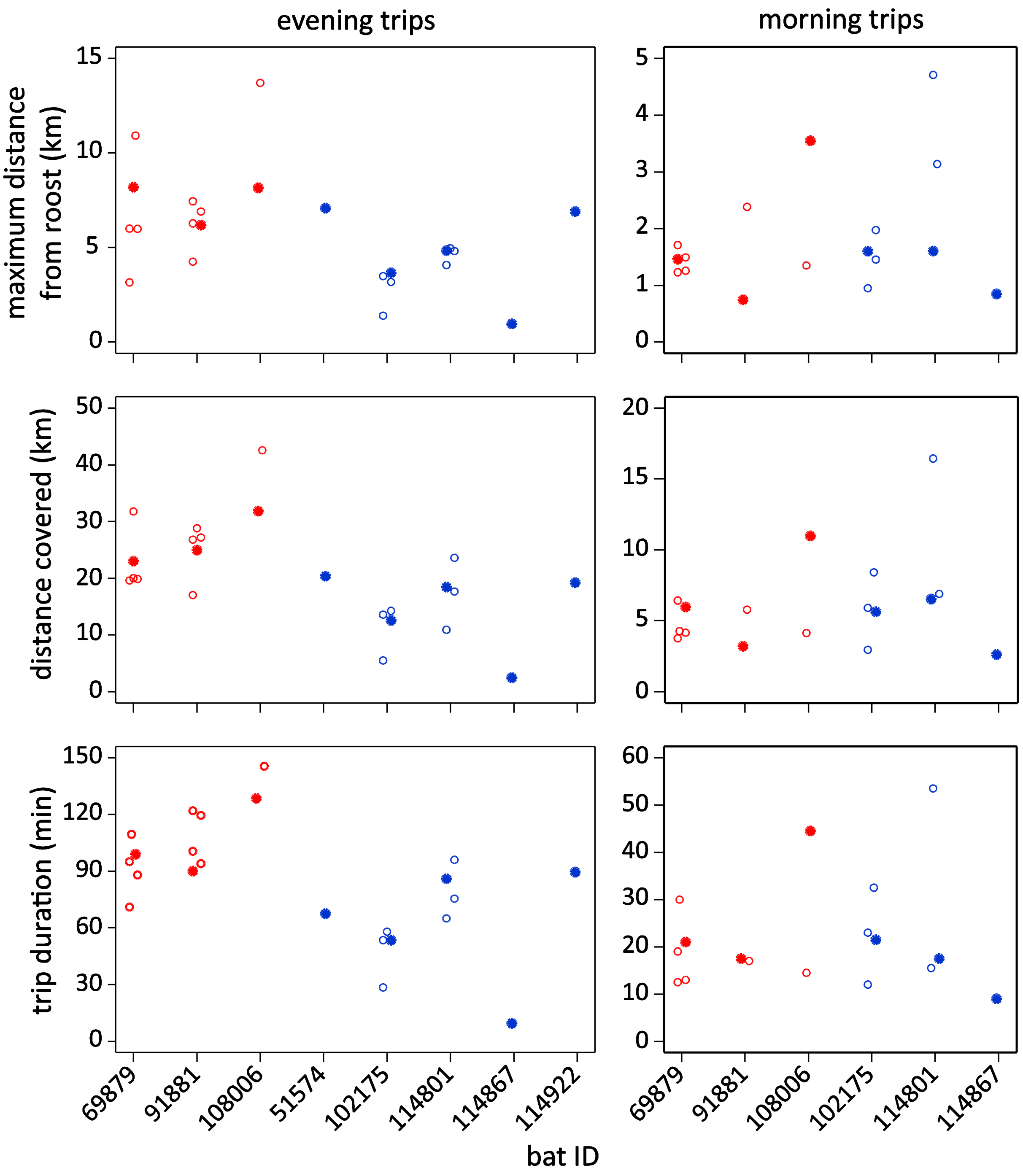

Supplementary Material Figure S1: Maximum distance from the roost, total distance covered, and duration of the respective trips (from top to bottom) for all 40 bat trajectories. The left column summarizes the data for evening trips, the right column for morning trips. Data of females is represented by red circles, data of males by blue circles. For every individual and daytime (i.e. evening or morning), one trip was randomly chosen for statistical analysis. These data points are represented by large, filled circles.

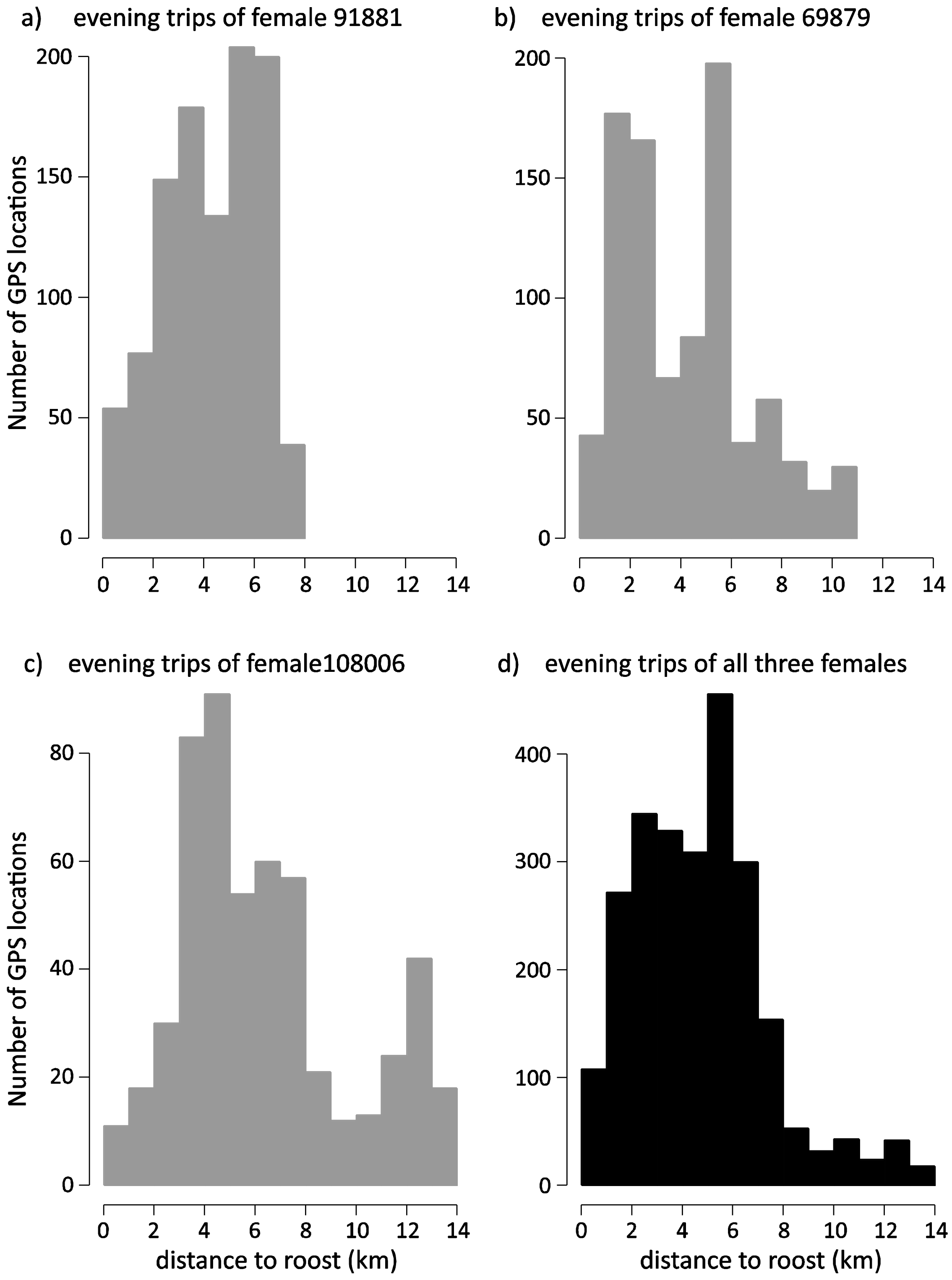

Supplementary Material Figure S2: Distance of GPS locations to the roost, pooled for all evening trips of the respective females (a-c), and for all evening trips off all three females together (d).

Supplementary Material Table S1: Summary of the bat presence model

Generalized linear mixed model fit by maximum likelihood (Laplace Approximation) ['glmerMod']
Family: binomial (logit)
Formular = bat_presence ~ (habitat + dist_wind_turbine + dist_linear_structure) * sex +
+ (1 | bat_ID/bat_trip) |

AIC	BIC	logLik	deviance	df.resid
53999.3	54163.1	-26979.6	53959.3	26554

Scaled residuals:

Min	1Q	Median	3Q	Max
-4.9469	-0.9796	-0.7583	-0.4902	12.0999

Random effects:

Groups	Name	Variance	Std.Dev.
bat_trip:bat_ID	(Intercept)	0.1017	0.3188
bat_ID	(Intercept)	0	0

26574 observations 40 groups: bat_trip:bat_ID

Fixed effect	Estimate	Std. Error	z value	P value	significance
conventional cropland	0.71352	0.10695	6.672	2.53E-11	***
forest	-0.07096	0.12183	-0.582	0.56029	
grasslands	1.58816	0.10945	14.511	2.00E-16	***
organic cropland	1.67785	0.10432	16.084	2.00E-16	***
bushes & hedges	0.6845	0.14433	4.742	2.11E-06	***
urban	1.37295	0.13726	10.003	2.00E-16	***
water	1.99024	0.12638	15.747	2.00E-16	***
distance to wind turbine	-0.69613	0.07711	-9.027	2.00E-16	***
distance to linear structure	-2.76779	0.12623	-21.926	2.00E-16	***
sex=male	-3.09939	0.19772	-15.676	2.00E-16	***
forest : sex=male	1.6025	0.08618	18.595	2.00E-16	***
grasslands : sex=male	1.59955	0.08801	18.174	2.00E-16	***
organic cropland : sex=male	1.23357	0.07124	17.315	2.00E-16	***
bushes & hedges : sex=male	1.63318	0.15807	10.332	2.00E-16	***
urban : sex=male	1.83522	0.16593	11.06	2.00E-16	***
water : sex=male	2.08202	0.11682	17.822	2.00E-16	***
distance to wind turbine : sex=male	2.3991	0.16049	14.948	2.00E-16	***
distance to linear structure : sex=male	-0.67354	0.24149	-2.789	0.00528	**

Significance codes: < 0.001 '***' 0.001 '**' 0.01 '*'

Supplementary Material Table S2: AICc values used for model selection. The full model explaining bat presence by distance to linear structures, distance to wind turbines, habitat type, and interactions of these variables was the best model and thus was used in this study.

Global model call: glmer(formular = bat_presence ~ (habitat + dist_wind_turbine + dist_linear_structure) * sex + (1 | bat_ID/bat_trip), data = bat_CRW, family = "binomial")

Model selection table

dist_linear_ structure	dist_wind_ turbine	habitat	sex	dist_linear_str: sex	dist_wt: sex	habitat:sex	df	logLik	AICc	delta AICc	weight
-2.768	-0.6961	+	+	+	+	+	20	-26979.65	53999.3	0	0.948
-2.954	-0.7072	+	+	NO	+	+	19	-26983.56	54005.1	5.81	0.052
-2.682	-0.1288	+	+	+	NO	+	19	-27095.51	54229.1	229.73	0
-2.663	NO	+	+	+	NO	+	18	-27097.43	54230.9	231.56	0
-3.021	-0.1183	+	+	NO	NO	+	18	-27108.63	54253.3	253.95	0
-2.999	NO	+	+	NO	NO	+	17	-27110.24	54254.5	255.18	0
NO	-0.5528	+	+	NO	+	+	18	-27377.63	54791.3	791.95	0
-2.427	-0.566	+	+	+	+	NO	14	-27469.78	54967.6	968.24	0
-3.084	-0.5867	+	+	NO	+	NO	13	-27512.72	55051.4	1052.11	0
NO	NO	+	+	NO	NO	+	16	-27523.39	55078.8	1079.46	0
NO	0.0769	+	+	NO	NO	+	17	-27522.69	55079.4	1080.08	0
-2.369	-0.1298	+	+	+	NO	NO	13	-27537.56	55101.1	1101.8	0
-2.35	NO	+	+	+	NO	NO	12	-27539.51	55103	1103.71	0
-3.092	-0.1108	+	NO	NO	NO	NO	11	-27592.29	55206.6	1207.26	0
-3.091	-0.1104	+	+	NO	NO	NO	12	-27591.37	55206.7	1207.41	0
-3.072	NO	+	NO	NO	NO	NO	10	-27593.72	55207.4	1208.11	0
-3.071	NO	+	+	NO	NO	NO	11	-27592.78	55207.6	1208.24	0
NO	-0.3897	+	+	NO	+	NO	12	-27958.37	55940.8	1941.42	0
NO	0.09852	+	+	NO	NO	NO	11	-28040.35	56102.7	2103.39	0
NO	NO	+	+	NO	NO	NO	10	-28041.49	56103	2103.66	0
NO	0.09807	+	NO	NO	NO	NO	10	-28041.56	56103.1	2103.79	0
NO	NO	+	NO	NO	NO	NO	9	-28042.69	56103.4	2104.05	0
-3.276	-1.347	NO	+	+	+	NO	8	-29376.41	58768.8	4769.5	0
-3.204	-0.8954	NO	+	+	NO	NO	7	-29448.06	58910.1	4910.79	0
-4.379	-1.421	NO	+	NO	+	NO	7	-29496.05	59006.1	5006.77	0
-3.065	NO	NO	+	+	NO	NO	6	-29558.97	59130	5130.62	0
-4.365	-0.8976	NO	+	NO	NO	NO	6	-29585.73	59183.5	5184.13	0
-4.37	-0.8844	NO	NO	NO	NO	NO	4	-29610.74	59229.5	5230.14	0
-4.218	NO	NO	+	NO	NO	NO	5	-29697.53	59405.1	5405.73	0
-4.224	NO	NO	NO	NO	NO	NO	3	-29715.65	59437.3	5437.98	0
NO	-1.112	NO	+	NO	+	NO	6	-30555.56	61123.1	7123.79	0
NO	-0.6315	NO	+	NO	NO	NO	5	-30635.76	61281.5	7282.2	0
NO	-0.6061	NO	NO	NO	NO	NO	3	-30660.77	61327.5	7328.21	0
NO	NO	NO	NO	NO	NO	NO	3	-30699.49	61405	7405.65	0
NO	NO	NO	+	NO	NO	NO	4	-30699.49	61407	7407.65	0
